# Vertical Graphene-Based Printed Electrochemical Biosensor for Simultaneous Detection of Four Alzheimer’s Disease Blood Biomarkers

**DOI:** 10.3390/bios13080758

**Published:** 2023-07-25

**Authors:** Mifang Li, Yu Zeng, Zhen Huang, Lingyan Zhang, Yibiao Liu

**Affiliations:** Longgang Central Hospital of Shenzhen, Shenzhen 518116, China; limifang01@163.com (M.L.); zykk@21cn.com (Y.Z.); huangzh1976@126.com (Z.H.)

**Keywords:** printed electrochemical biosensor, Alzheimer’s disease, biomarkers, vertical graphene

## Abstract

Early detection and timely intervention play a vital role in the effective management of Alzheimer’s disease. Currently, the diagnostic accuracy for Alzheimer’s disease based on a single blood biomarker is relatively low, and the combined use of multiple blood biomarkers can greatly improve diagnostic accuracy. Herein, we report a printed electrochemical biosensor based on vertical graphene (VG) modified with gold nanoparticles (VG@nanoAu) for the simultaneous detection of four Alzheimer’s disease blood biomarkers. The printed electrochemical electrode array was constructed by laser etching and inkjet printing. Then gold nanoparticles were modified onto the working electrode surface via electrodeposition to further improve the sensitivity of the sensor. In addition, the entire printed electrochemical sensing system incorporates an electrochemical micro-workstation and a smartphone. The customized electrochemical micro-workstation incorporates four electro-chemical control chips, enabling the sensor to simultaneously analyze four biomarkers. Consequently, the printed electrochemical sensing system exhibits excellent analytical performance due to the large surface area, biocompatibility, and good conductivity of VG@nanoAu. The detection limit of the sensing system for Aβ40, Aβ42, T-tau, and P-tau181 was 0.072, 0.089, 0.071, and 0.051 pg/mL, respectively, which meets the detection requirements of Alzheimer’s disease blood biomarkers. The printed electrochemical sensing system also exhibits good specificity and stability. This work has great value and promising prospects for early Alzheimer’s disease diagnosis using blood biomarkers.

## 1. Introduction

Alzheimer’s disease (AD) is characterized as a chronic neurodegenerative disorder with a very long duration cycle. AD patients exhibit symptoms such as memory impairment and language dysfunction, which can lead to death in severe cases, thereby imposing a huge economic and mental burden on the country, society, and the patient’s family [1,2]. However, currently, the pathogenesis of AD remains unclear, and no effective drugs to cure the disease have been developed. Thus, early diagnosis, detection and intervention are particularly important. In clinical settings, AD is diagnosed by cognitive scales, neuroimaging and molecular biomarkers in the cerebrospinal fluid (CSF) [2,3,4]. Among them, changes in the level of molecular biomarkers in the CSF can directly reflect neurological damage in the brain, thereby allowing for earlier diagnosis of AD. However, CSF is difficult to obtain, and is highly invasive and prone to adverse reactions, which greatly limits its application in large-scale general screening. Therefore, exploring diagnostic methods for AD in terms of easily accessible blood biomarkers is important.

Recently, numerous studies have found that blood biomarkers, including Aβ40, Aβ42, T-tau, P-tau181, etc., are closely associated with the onset of AD, thereby indicating the feasibility of diagnosing AD [5,6,7]. However, the levels of AD blood biomarkers are extremely low, reaching the picogram per milliliter (pg/mL) range [8], which surpasses the detection limit of the conventional enzyme-linked immunosorbent assay (ELISA) technique. Therefore, developing ultrasensitive blood detection methods is extremely urgent. At present, several techniques have been developed to analyze blood biomarkers for AD diagnosis, including electrochemical [9,10,11], fluorescence [12,13,14], field effect transistor [15,16], surface-enhanced Raman scattering (SERS) [17,18], and colorimetric methods [19,20]. Among these approaches, the electrochemical analytical method exhibits immense promise in disease diagnosis owing to its exceptional sensitivity and ease of miniaturization.

Printed sensors are made on substrates using printing technology (e.g., inkjet printing, screen printing, etc.) [21,22,23]. Given their advantages of low cost, large-scale production, and convenient customization, printed sensors are widely used in disease diagnosis [24,25]. In the field of AD blood biomarker detection, there have been some reports, such as Moreira’s screen-printing electrochemical sensor for the ultra-sensitive detection of Aβ42 [26], Vu et al.’s microelectrode-based SERS sensor for the detection of Aβ [17], and Subramaniyan Parimalam et al.’s laser-printed microfluidic chip sensor for the detection of tau protein [27]. 

Currently, most detection methods only detect one type of AD blood biomarker. However, individual biomarkers greatly vary among individuals due to genetic factors, dietary habits, living environment, and other factors, thereby resulting in low AD diagnosis accuracy based on a single biomarker. AD diagnosis accuracy can be improved to some extent by combining multiple blood biomarkers [28,29]. In previous studies, we reported an electrochemical biosensor based on superwetting microdroplet array for multiple AD blood marker detection. The sensor displayed good analytical performance, but in subsequent usage we discovered that the shedding of hydrophobic molecules led to the disruption of the microdroplet array, resulting in poor durability of the entire sensor [30]. Based on this, we attempted to construct an electrode array by printing technology instead of using superwetting microarray technology to avoid the issue of easy shedding of hydrophobic molecules. Considering the advantages of easy miniaturization and mass production, combining electrochemical and printed sensing techniques to construct multiplexed printed electrochemical sensors to simultaneously detect multiple AD biomarkers holds great value.

**Figure 1:**
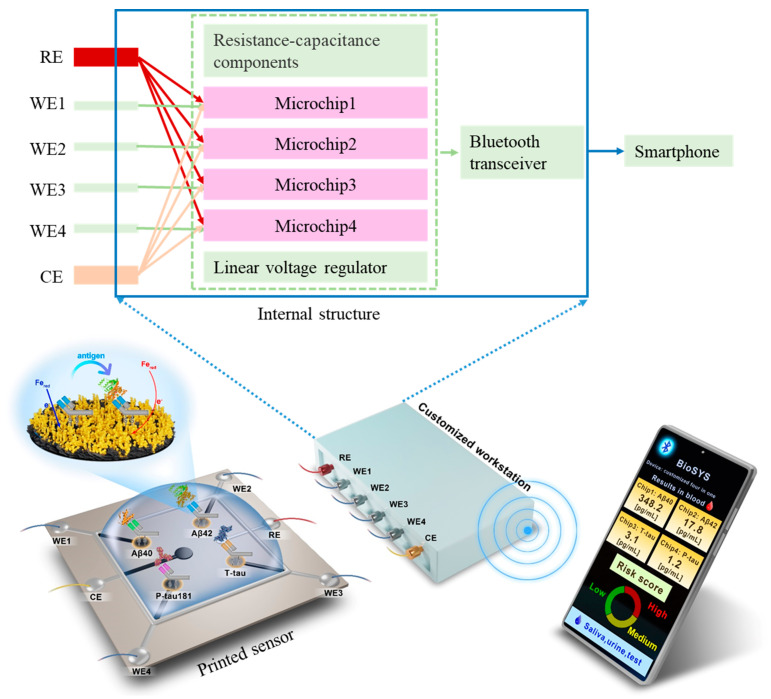
Schematic representation of the printed electrochemical sensing system for simultaneous detection of four AD biomarkers.

In this work, we develop a printed electrochemical biosensor based on vertical graphene modified with gold nanoparticles (VG@nanoAu) by combining electrochemistry with printed sensor technology for the ultrasensitive detection of four AD biomarkers. As shown in Figure 1, in this printed electrochemical sensing system, there are four working electrodes (WE1, WE2, WE3, and WE4), one reference electrode (RE), and one counter electrode (CE). The working electrode material is VG@nanoAu, and the counter electrode material is VG. The modification of nanogold on VG by electrodeposition is to increase the electroactive area, thereby improving the sensitivity of the sensor. The VG@nanoAu surface is functionalized with pretreated antibodies. The antibody of the target protein is treated with 2-mercaptoethylamine (2-MEA), resulting an antibody with thiol termination, which allows it to be immobilized to the electrode surface by gold-sulfur bond [31]. After the binding of the target antigen and antibody, the impedance on the working electrode surface increases, leading to a decrease in the differential pulse voltammetry (DPV) signal. The change in the DPV signal is directly proportional to the concentration of the target antigen. Therefore, the concentration of the target antigen can be calculated according to the variation in the DPV signal. In particular, a specially customized electrochemical micro-workstation is employed to control and monitor the electrochemical signal. As shown in the internal structure, the micro-workstation incorporates four electrochemical control chips. These four chips are independent of each other, which enables the sensor to simultaneously analyze four biomarkers. The printed electrochemical sensing system demonstrates promising prospects in early AD diagnosis based on multiple blood biomarkers.

## 2. Materials and Methods

### 2.1. Chemicals and Reagents

T-tau protein, P-tau181 protein, and Aβ antibody were obtained from Abcam Ltd. (Hong Kong, China). Aβ protein, K_3_[Fe(CN)_6_]/K_4_[Fe(CN)_6_], glucose (GLU), bovine serum albumin (BSA), potassium chloride (KCl), phosphate-buffered solution (PBS, pH = 7.4, 10 mM), 2-mercaptoethylamine, and chloroauric acid (HAuCl_4_) were purchased from Sigma-Aldrich (Shanghai, China). The antibodies of T-tau and P-tau181 were purchased from Thermo Fisher Scientific Co., Ltd. (Beijing, China). All chemical reagents used were of high purity grade. All solution preparations were made using ultrapure water (Milli-Q, 18.2 MΩ).

### 2.2. Characterization and Measurement

Field-emission scanning electron microscopy (SEM, Thermo Fisher, FEI Apreo S, Waltham, MA, USA) was employed to analyze the surface morphology and elemental distribution of VG@nanoAu. The VG array was fabricated by laser etching (Laser engraving machine, SCM-2200, Wuhan Sangong Laser Technology Co. Ltd., Wuhan, China), and the Ag/AgCl reference electrode and Ag conductor were constructed using a Fujifilm Dimatix nanomaterial deposition jet printer (DMP2850). Electrochemical tests were conducted utilizing a tailor-made electrochemical micro-workstation (Refresh AI Biosensor Co., Ltd., Shenzhen, China). 

### 2.3. Construction of Printed Electrode Array Based on VG@nanoAu

**Figure 2:**
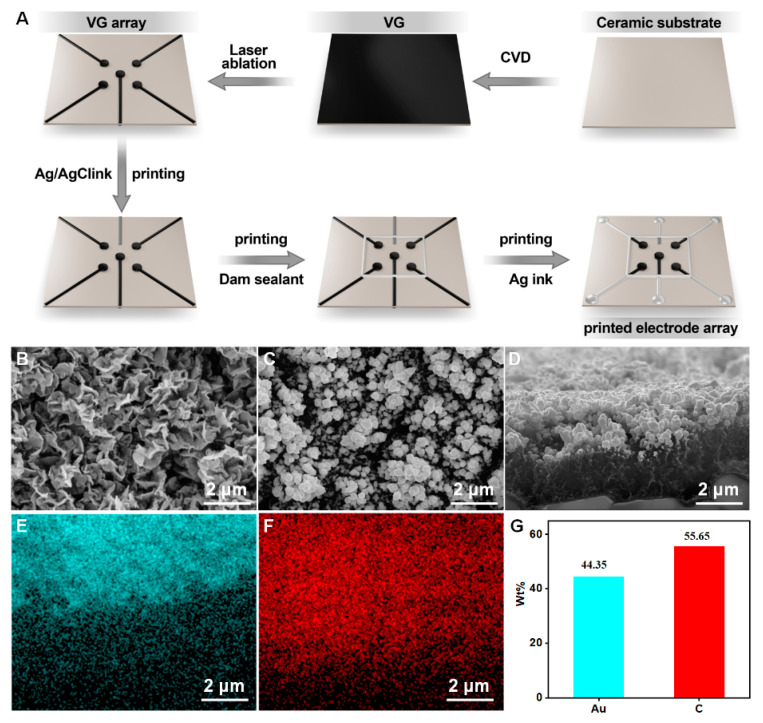
(**A**) The preparation process of printed electrode array. (**B**) The surface morphology of VG. (**C**,**D**) Surface and cross-section view SEM images of VG@nanoAu. (**E**–**G**) Surface element distribution of VG@nanoAu.

First, a chemical vapor deposition (CVD) was used to fabricate a VG layer on a ceramic substrate (1 cm × 1 cm). Then, nanogold is deposited onto the surface of the VG by electrodeposition of 10 mM chloroauric acid, as previously reported. Afterward, the VG is laser-etched into an array as shown in Figure 2A. Subsequently, a silver/silver chloride ink is printed onto the substrate surface as a reference electrode. Then, a dam sealant is printed as a reaction cell. Finally, the silver ink is printed onto the surface as wires. In addition, insulating ink is printed on the periphery as an enclosure. After the completion of the VG electrode array, gold nanoparticles were deposited onto the VG working electrodes by electrodeposition in a solution of 10 mM HAuCl_4_. The deposition voltage and deposition time is −1.8 V and 300 s. At this point, the entire printed electrode array based on VG@nanoAu was constructed.

### 2.4. Preparation of Printed Electrochemical Sensor Based on VG@nanoAu for AD Biomarkers

After the printed electrode array is completed, the printed electrochemical sensor is constructed through a series of modifications. First, a volume of 3 μL antibody solution was dropped onto the VG@nanoAu electrode surface and allowed to incubate at 37 °C for 1 h. Beforehand, the antibody of target protein was treated with 2-mercaptoethylamine, resulting in an antibody with thiol termination. Then, a blocking solution containing 3 μL of BSA (1%) was applied to prevent nonspecific binding (the treatment time of BSA is 1 h at 37 °C), followed by the addition of a protein mixture consisting of Aβ40, Aβ42, T-tau, and P-tau181. The reaction mixture was then incubated at 37 °C for 1 h. Following each modification step, the electrode surface is rinsed three times with PBS buffer solution. Finally, the printed electrode array is combined with a customized electrochemical micro-workstation. A printed electrochemical sensing system for detecting four AD biomarkers is successfully constructed.

The concentration of antigen was measured using a customized electrochemistry micro-workstation through DPV. DPV test was conducted in 5 mM [Fe(CN)_6_]^3−^/[Fe(CN)_6_]^4−^ solution containing 0.1 M KCl. The scan rate is 0.1 V/s. the pulse width and period are 0.05 s and 0.5 s, respectively. The amplitude is 0.05 V. The working voltage range of DPV signals was 0–0.4 V. Then, the peak current change before and after the binding of the target protein with the antibody was measured, and the antigen concentration was determined using this signal.

The selectivity of this printed electrochemical sensor based on VG@nanoAu for detecting four AD blood biomarkers was assessed in PBS solutions containing Aβ40, Aβ42, Tau441, P-tau181, HSA, and GLU.

### 2.5. The Application of This Printed Electrochemical Sensor in Clinical Samples

Two clinical samples were tested using the sensor to validate its clinical application value. The clinical samples were obtained from Longgang Central Hospital of Shenzhen, and the study was approved by the Ethics Committee of the Longgang Central Hospital of Shenzhen (protocol code 2021ECYJ048 and date of approval 2 September 2021). Briefly, a volume of 3 μL antibody solution was dropped on the working electrode surface. Then, 3 μL blocking solution (1% BSA) was applied to prevent nonspecific binding after 1 h of incubation. At this point, the initial current was tested by DPV, and the DPV signal was recorded. Then, 20 μL of serum sample (diluted with PBS solution at 1:3) was added to the sample pool and incubated at 37 °C for 1 h. The variation in peak current before and after incubation was monitored and utilized for the calculation of the antigen concentration. 

## 3. Results and Discussion

### 3.1. Preparation and Characterization of Printed Electrode Array

The construction process of the printed VG electrode array is shown in Figure 2A. First, VG was grown on a ceramic substrate by chemical vapor deposition, then it was etched into an electrode array using laser ablation. Afterward, the reference electrode and electrode wires were printed using an inkjet printer, resulting in a VG-based electrode array. After the construction of the VG electrode array, gold nanoparticles were deposited onto the surface of the VG working electrode to further enhance the sensitivity of the printed electrochemical sensor. The detailed procedures are described in the experimental section. The surface morphology of the VG was characterized, and Figure 2B demonstrates the presence of a distinct flaky structure. After gold plating, the morphology is shown in Figure 2C. SEM images show that spherical gold particles appeared on the surface of the VG, and the size of gold particles is in a range of tens to a hundred nanometers. The cross-sectional images of VG@nanoAu demonstrated that the majority of gold nanoparticles were observed to be concentrated on the upper part region of the VG. (Figure 2D) The height of the VG is approximately 2 μm, and the thickness of the gold nanoparticles is approximately 1 μm. Surface element distribution was also characterized. As shown in Figure 2E–G, most areas of the VG surface are covered by gold nanoparticles, and the surface mass fraction of the gold element is 44.35%.

### 3.2. Construction and Analytical Performance of Printed Electrochemical Biosensor 

**Figure 3:**
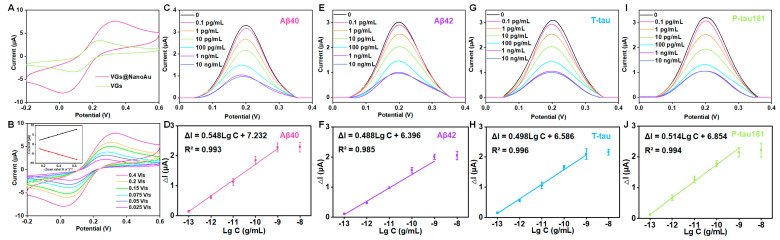
Analytical performance of the VG@nanoAu-based printed sensor for detecting four AD blood biomarkers. (**A**) CVs of VG and VG@nanoAu electrode. (**B**) CVs of the VG@nanoAu−based printed sensor at various scan rates. The inset graph illustrates the correlation between the peak current of CV and the square root of the scan rate. DPV signals toward four AD blood biomarkers and the linear relationship between peak current change value (ΔI) of DPV signals and the logarithm of antigen concentration of Aβ40 (**C**,**D**), Aβ42 (**E**,**F**), T−tau (**G**,**H**), and P−tau181 (**I**,**J**). CV and DPV test was conducted in 5 mM [Fe(CN)_6_]^3−^/[Fe(CN)_6_]^4−^ solution containing 0.1 M KCl at 0.1 V/s.

The electroactive area of VG and VG@nanoAu surfaces were compared using cyclic voltammetry (CV). The electroactive surface area was remarkably increased after being modified with gold nanoparticles (Figure 3A). In this process of CV, when an anodic potential is applied, ferrous ions undergo oxidation, resulting in the appearance of an oxidation peak. Conversely, when a cathodic potential is applied, ferric ions undergo reduction, leading to the manifestation of a reduction peak. Furthermore, the surface kinetics processes were assessed by conducting CV measurements at various scan rates. The result showed that, as depicted in Figure 3B, the ratio between the cathodic peak current (I_pc_) and anodic peak current (I_pa_) is greater than 1, and this ratio is also influenced by the scan rate, indicating that the electron transfer reaction is quasi-reversible. The peak current exhibited a linear correlation with the square root of the scan rate, which suggested that a diffusion-limited mass transfer process occurred at the working electrode interface. After modification with gold nanoparticles, the antibodies for the target protein were immobilized on the surface of the working electrode through gold–sulfur bonds. DPV peak current decreased after the modification of antibodies, thereby indicating successful antibody immobilization on the electrode surface. The antibody concentration used in the experiment is 10 μg/mL according to our previous study [30,32]. Subsequently, BSA was employed to obstruct unbound sites, resulting in a further reduction of the DPV peak current (Figure S1). Finally, a mixture of four proteins (Aβ40, Aβ42, T-tau, and P-tau181) was pipetted into the sample well and incubated for 1 h, and the DPV signals before and after incubation were recorded. At this point, the printed electrochemical sensor for detecting four AD blood biomarkers had been successfully constructed. 

Furthermore, as illustrated in Figure 3C–J, as the antigen concentration increases, the DPV peak current signal gradually decreases. A logarithmic linear correlation is observed between the change in peak current value (ΔI) and the target protein concentration. The detection limits of the printed electrochemical sensor for Aβ40, Aβ42, T-tau, and P-tau181 were 0.072, 0.089, 0.071, and 0.051 pg/mL, respectively. The detection limit was calculated according to three times the standard deviation of the blank sample [33,34]. Briefly, PBS buffer was used as a blank sample, which was measured ten times with the printed sensors. Then, the standard deviation was calculated. The detection limit is calculated by bringing the standard deviation into the standard curve equation. (The standard curve equations are shown in Figure 3). In physiological conditions, the concentrations of AD blood biomarkers, such as Aβ40, Aβ42, T-tau, and P-tau181 are measured at pg/mL levels. Our developed VG@nanoAu-based printed sensor showed excellent analytical performance with detection limits below 0.1 pg/mL, thereby meeting the requirement for ultra-sensitive detection of AD biomarkers in blood samples. 

### 3.3. Selectivity and Stability

**Figure 4:**
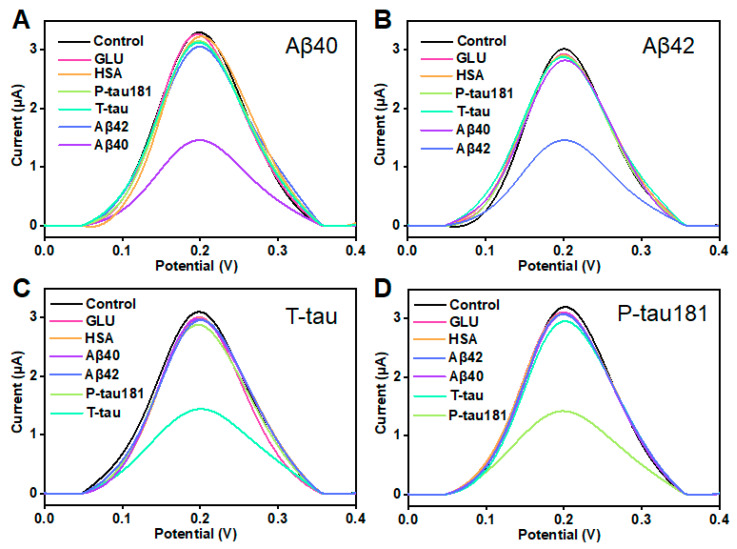
Selectivity of the printed electrochemical biosensor based on VG@nanoAu. (**A**) DPV signals for Aβ40 and Aβ42, T-tau, P-tau181, GLU, and HSA. The concentration of Aβ40 is 100 pg/mL, and other protein concentration is 10 ng/mL. (**B**) DPV signals for 100 pg/mL Aβ42 and 10 ng/mL other protein including Aβ40, T-tau, P-tau181, GLU, and HSA. (**C**) DPV signals for 100 pg/mL T-tau and 10 ng/mL other protein including Aβ40, Aβ42, P-tau181, GLU, and HSA. (**D**) DPV signals for 100 pg/mL P-tau181 and 10 ng/mL other protein including Aβ40, Aβ42, T-tau, GLU, and HSA.

In clinical applications, selectivity and stability are considered as two critical parameters in evaluating a sensor. This study also investigated the selectivity and stability of this printed sensor. According to the illustration in Figure 4A, when the concentration of Aβ40 was 10 pg/mL, the peak current significantly decreased. When 100-fold of other proteins including Aβ42, T-tau, P-tau181, GLU, and HSA was added, the variation in peak current is insignificant, and was much lower than the ΔI of Aβ40. The result showed that the printed electrochemical sensor has good selectivity for Aβ40. Similarly, this printed sensor showed excellent selectivity for Aβ42, T-tau, and P-tau181 (Figure 4B–D). In addition, this result indicates that the four target proteins (Aβ40, Aβ42, T-tau, and P-tau181) do not interfere much with each other. Furthermore, the stability of this printed electrochemical sensor was assessed by testing the 100 pg/mL target proteins (Aβ40, Aβ42, T-tau, and P-tau181) six times over 2 weeks. During this period, the printed sensor was kept in a refrigerator at 4 °C. As shown in Figure S2, the ΔI maintained more than 90% of the initial value even after 14 days, which suggested that this VG@nanoAu-based printed sensor had good stability. These results also demonstrated that this VG@nanoAu-based printed electrochemical exhibited outstanding stability and specificity. 

### 3.4. Application of This Printed Electrochemical Sensor in Clinical Samples

To further assess its potential for clinical application, we conducted measurements of Aβ40, Aβ42, T-tau, and P-tau181 in two clinical samples using the printed electrochemical sensor, and the results were compared with those obtained from the classic ELISA method. The results are shown in Table 1. The detection results of Aβ obtained from this printed electrochemical sensor showed no significant difference compared to the results obtained from the classic ELISA method. Moreover, this printed sensor can accurately measure the levels of tau proteins in the two clinical samples. However, the classic ELISA method cannot detect extremely low levels of tau and P-tau181. These results demonstrate the printed electrochemical sensor holds great potential in clinical applications.

Finally, the analytical performance of this printed electrochemical sensor was compared to that of previously reported methods. Among the numerous reports on detecting AD biomarkers, the majority of studies have focused on the detection of a single AD biomarker. Some studies that detect multiple AD biomarkers are separately listed and compared with our method, as shown in Table 2. For instance, an electrochemical device based on screen-printed electrodes developed by Sanati-Nezhad’s group [35] simultaneously detects two proteins, C-Tau and NFL. The detection limits for C-Tau and NFL are 0.32 pg/mL and 0.18 pg/mL, respectively, with a linear range from 10 pg/mL to 100 ng/mL. In comparison, our sensor exhibits a linear range of 0.1 pg/mL to 1 ng/mL. Under physiological conditions, the concentrations of most AD biomarkers such as Aβ, T-tau, P-tau, etc., fall within this range. This linear range of our printed sensor better meets the requirements for detecting clinical samples. For fluorescence detection, Chan et al. [36] developed a fluorescent sensor capable of simultaneously detecting three biomarkers, Aβ42, tau441, and p-tau181, with detection limits of 340.07, 669.44, and 493.79 pg/mL, respectively. This sensor meets the detection requirements for these biomarkers in cerebrospinal fluid but may face challenges when detecting biomarkers in blood samples. In addition, Kim et al. [37] designed an SPR biosensor based on gold nanocrystals with different morphologies for simultaneous detection of three biomarkers, Aβ40, Aβ42, and T-tau. This sensor requires the preparation of different gold nanocrystals and the specific modification of target protein antibodies, making the process relatively intricate. Our printed electrochemical sensor is fabricated through integrated inkjet printing technology, offering a simple and scalable approach for mass production. A specially customized electrochemical micro-workstation, incorporating four electrochemical control micro-chips, was utilized, enabling our detection platform to simultaneously analyze four biomarkers instead of sequentially testing them one by one. In summary, our printed sensor not only demonstrates outstanding analytical performance but also boasts a straightforward fabrication process that allows for efficient mass production.

## 4. Conclusions

In conclusion, we designed and constructed a VG@nanoAu-based printed sensor based on combining laser etching and inkjet printing technology for the simultaneous detection of four AD blood biomarkers. The decoration of the VG electrode surface with gold nanoparticles remarkably increased the electroactive surface area and provided antibodies binding sites. In this printed electrochemical sensing system, a particularly customized electrochemical micro-workstation incorporating four independent control chips was employed to control and acquire electrochemical signals, which enables the sensor to simultaneously detect four biomarkers. The printed biosensor displayed outstanding detection performance. Consequently, the detection limits of the printed sensing system for Aβ40, Aβ42, T-tau, and P-tau181, were 0.072, 0.089, 0.071, and 0.051, respectively. In clinical sample detection, the printed electrochemical sensing system also demonstrated outstanding detection results. Therefore, this work provides a new detection method for early diagnosis of AD based on multiple blood biomarkers, and has broad application prospects in the field of combined diagnosis of multifactorial diseases.

## Figures and Tables

**Table 1:** Comparison between our printed electrochemical sensing system and typical ELISA for detecting four AD biomarkers in two clinical samples.

Sample	Biomarkers	Average Measured Concentration (pg/mL)	
		This Printed Sensor	ELISA
1	Aβ40	155 ± 10	153 ± 6.8
	Aβ42	15.4 ± 1.3	15.1 ± 1.2
	T-tau	4.8 ± 0.2	/
	P-tau181	1.8 ± 0.1	/
2	Aβ40	348 ± 16	350 ± 8.0
	Aβ42	17.8 ± 1.2	17.6 ± 0.9
	T-tau	3.1 ± 0.1	/
	P-tau181	1.2 ± 0.2	/

**Table 2:** A comparison of detection performance for multiple AD biomarkers.

Methods	Biomarkers	Detection limit	References
Electrochemistry	Aβ40, Aβ42, Tau, P-tau181,	2.20, 2.13, 2.45, 2.72 fM	[37]
Electrochemistry	T-tau, NFL	0.32, 0.18 pg/mL	[35]
Electrochemistry	Aβ, Tau, ApoE4,	8.6 × 10^−12^; 7.1 × 10^−11^; 5.91 × 10^−11^ mg/mL	[38]
Electrochemistry	Aβ40, Aβ42, T-tau, P-tau181	0.142, 0.176, 0.125, 0.089 pg/mL	[34]
Fluorescence	Aβ42, tau441, P-tau181	340.07, 669.44, 493.79 pg/mL	[36]
LSPR	Aβ40, Aβ42, T-tau,	34.9, 26, 23.6 fM	[39]
SERS	Aβ42 oligomers, Tau	3.7 × 10^−2^ nM 4.2 × 10^−4^ pM	[40]
IME sensor	Aβ40, Aβ42, T-tau	50.19, 143.44, 12.19 fM	[29]
Electrochemistry	Aβ40, Aβ42, T-tau, P-tau181	0.064, 0.012, 0.039, 0.041 pg/mL	[30]
Electrochemistry	Aβ40, Aβ42, T-tau, P-tau181	0.072, 0.089, 0.071, 0.051 pg/mL	This work

## Data Availability

The data presented in this study are available on request from the corresponding author.

## Supplementary Materials

The following supporting information can be downloaded at: https://www.mdpi.com/article/10.3390/bios13080758/s1, Figure S1: The characterization of modification processes of electrode surface.; Figure S2: Stability of the printed electrochemical sensor for detecting four AD biomarkers.
