# Supplementary material for: Vertical Graphene-Based Printed Electrochemical Biosensor for Simultaneous Detection of Four Alzheimer’s Disease Blood Biomarkers
Source: Biosensors (Basel). 2023 Jul 25;13(8):758. doi: 10.3390/bios13080758 (PMC10452345; doi:10.3390/bios13080758)
Supplement: Supplementary file 1 [file biosensors-13-00758-s001.zip › biosensors-2486664-supplementary.pdf]

Supplementary Materials

Vertical Graphene-Based Printed Electrochemical Biosensor for Simultaneous Detection of Four Alzheimer's Disease Blood Biomarkers

Mifang Li †, Yu Zeng †, Zhen Huang, Lingyan Zhang* and Yibiao Liu*

Longgang Central Hospital of Shenzhen, Shenzhen 518116, China

* Correspondence: 18819818005@163.com (L.Z.); liuyibiao12345@126.com (Y.L.)

† These authors contributed equally to this work.

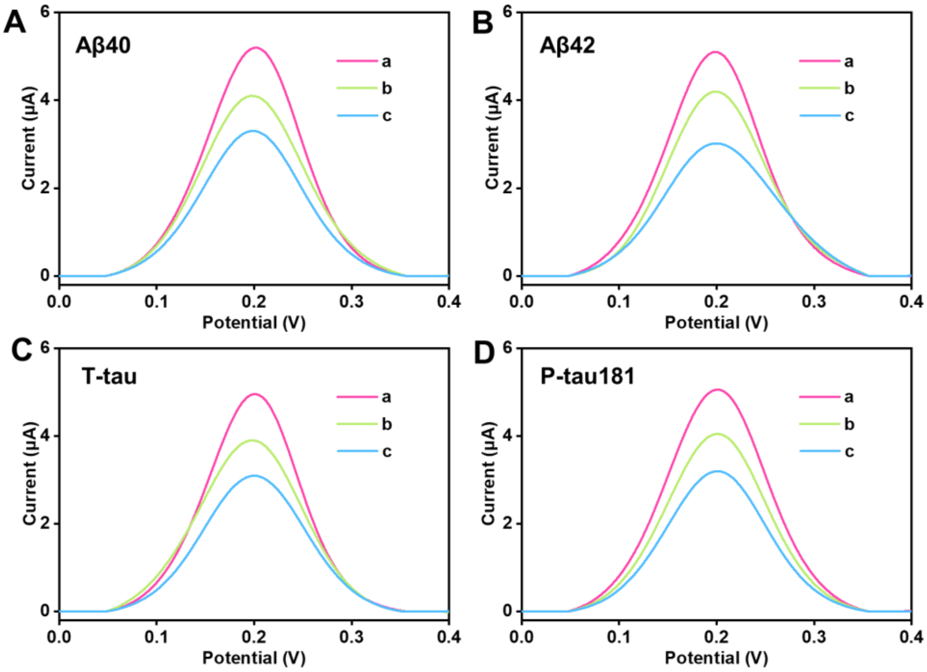

Figure S1. The characterization of modification processes of electrode surface. (A)DPV signals of the VG@nanoAu working electrode (curve a) modified with Aβ40 antibody (curve b), Aβ40 antibody + BSA (curve c). (B) DPV signals of the VG@nanoAu working electrode (curve a) modified with antibody of Aβ42 antibody (curve b), Aβ42 antibody + BSA (curve c). (C) DPV signals of the VG@nanoAu working electrode (curve a) modified with T-tau antibody (curve b), T-tau antibody + BSA (curve c). (D)DPV signals of the VG@nanoAu working electrode (curve a) modified with P-tau181 antibody (curve b), P-tau181 antibody + BSA (curve c). The DPVs are carried out in 5 mM $[Fe(CN)_6]^{3-}/[Fe(CN)_6]^{4-}$ solution containing 0.1 M KCl at 0.1 V/s.

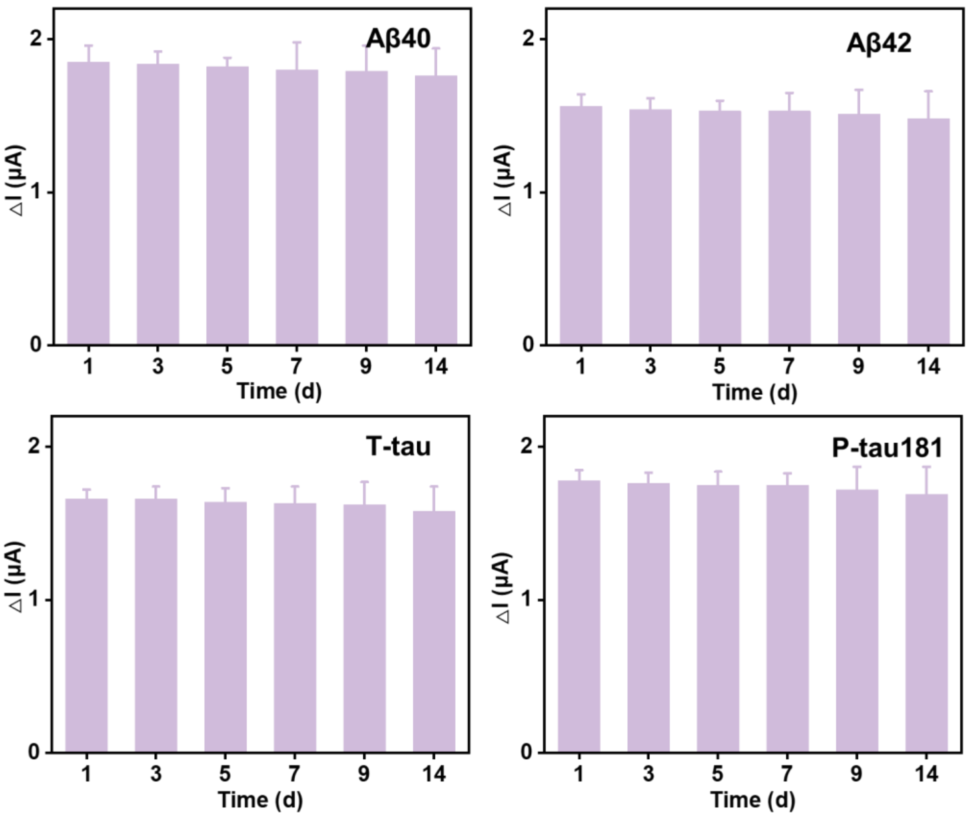

Figure S2. Stability of the printed electrochemical sensor for detecting four AD biomarkers.
